# Role of TP53 Mutations and EGFR Amplification in Risk Stratification of Early‐Stage EGFR‐Mutated Non‐Small Cell Lung Cancer With Immunohistochemistry as a Surrogate Marker

**DOI:** 10.1111/1759-7714.70058

**Published:** 2025-04-01

**Authors:** Meejeong Kim, Gyeong Sin Park, Kyo Young Lee, Seok Whan Moon, Yeoun Eun Sung

**Affiliations:** ^1^ Department of Hospital Pathology, Seoul St. Mary's Hospital, College of Medicine The Catholic University of Korea Seoul Republic of Korea; ^2^ Department of Thoracic and Cardiovascular Surgery, Seoul St. Mary's Hospital, College of Medicine The Catholic University of Korea Seoul Republic of Korea

**Keywords:** early‐stage lung cancer, EGFR amplification, EGFR mutation, non‐small cell lung cancer (NSCLC), TP53 mutation

## Abstract

**Background:**

Non‐small cell lung carcinoma (NSCLC) is a leading cause of cancer‐related mortality, with recurrence risks posing significant challenges in early‐stage disease management. While epidermal growth factor receptor (EGFR) mutations are common, the role of concurrent genetic alterations remains underexplored, and findings have often been inconsistent, particularly in early‐stage tumors.

**Methods:**

We retrospectively analyzed 424 EGFR‐mutated NSCLC patients diagnosed from 2017 to 2022. Next‐generation sequencing (NGS) was used to identify genetic alterations, and immunohistochemistry (IHC) was employed to correlate TP53 mutations and EGFR amplification with protein expression. Survival outcomes were assessed using Kaplan–Meier and Cox regression analyses, while predictive cutoffs were determined with receiver operating characteristic (ROC) curve analysis.

**Results:**

TP53 mutations and EGFR amplification were more prevalent in Stages 2–4 compared to Stage 1 (*p* < 0.001 and 0.005, respectively). In Stage 1, TP53 mutations, particularly exon 4 and frameshift/nonsense types, were associated with worse overall survival (OS) and disease‐free survival (DFS). EGFR amplification was linked to shorter DFS in Stage 1 (*p* = 0.006). Both alterations correlated with aggressive pathological features, including advanced N stage, lymphovascular invasion, and high histological grade. IHC cutoffs of 15% for TP53 and *H*‐score ≥ 180 for EGFR amplification demonstrated high predictive accuracy (AUC = 0.981 and 0.936, respectively).

**Conclusion:**

Specific subtypes of TP53 mutations and EGFR amplification are important prognostic markers in early‐stage NSCLC. IHC offers a practical surrogate for genetic testing, aiding in risk stratification and guiding adjuvant therapy decisions for high‐risk patients. Larger validation studies are warranted.

## Graphical abstract

Specific TP53 mutations and epidermal growth factor receptor (EGFR) amplification are associated with worse prognosis in early‐stage EGFR‐mutated non‐small cell lung cancer (NSCLC). Immunohistochemistry (IHC) (p53, EGFR) correlates with genetic alterations, providing a practical surrogate marker for risk stratification.

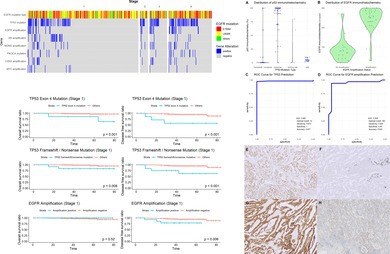

## 1 Introduction

Non‐small cell lung cancer (NSCLC) remains a leading cause of cancer‐related mortality worldwide [1]. Epidermal growth factor receptor (EGFR) mutations are observed in approximately 20% of lung adenocarcinomas [2], with higher prevalence among Asian patients, reaching nearly 50% [3]. Tumors harboring activating EGFR mutations exhibit sensitivity to EGFR‐tyrosine kinase inhibitors (TKIs) [4, 5]; however, treatment outcomes are not uniformly favorable. The clinical course of EGFR‐mutated NSCLC is influenced by a range of factors, including smoking history, histological subtype, EGFR mutation type, metastatic status, and patient performance status [6, 7, 8, 9].

The widespread adoption of next‐generation sequencing (NGS) has expanded the understanding of genetic alterations beyond EGFR mutations. Previous studies have primarily focused on the impact of concurrent mutations in advanced NSCLC and their influence on TKI responsiveness. For instance, TP53 mutations are associated with reduced TKI efficacy [7] and, along with ERBB2 or MET amplification, are linked to shorter progression times on EGFR‐TKI therapy [9]. TP53 mutations also serve as independent markers of shorter overall survival (OS) in advanced NSCLC [10]. While no significant association has been established between TP53 mutations and treatment response duration [10]. Nonetheless, a trend toward shorter progression‐free survival (PFS) has been reported in patients receiving erlotinib [10].

In early‐stage NSCLC, where standard treatment involves surgical resection, recurrence rates remain high, with 30%–55% of patients experiencing relapse within 5 years [11]. Adjuvant osimertinib has demonstrated significant OS benefits in resected, EGFR‐mutated Stage IB–IIIA NSCLC [12]. Identifying patients at high risk of recurrence, particularly in early‐stage disease, is critical. This study explores the clinical significance, prognostic implications, and risk factor correlations of concurrent genetic alterations in early‐stage EGFR‐mutated NSCLC.

## 2 Methods

### 2.1 Study Design and Patient Population

Between April 2017 and March 2022, a total of 3077 solid malignancy cases underwent NGS molecular testing at Seoul St. Mary's Hospital. Among them, 1105 cases were diagnosed as non‐small cell lung cancer (NSCLC). After excluding 10 patients who received neoadjuvant therapy and 21 patients with recurrent NSCLC, 1074 newly diagnosed NSCLC cases were identified. Of these, 424 patients with EGFR‐positive tumors were included in this study. Clinical data and follow‐up information were extracted from hospital records. Histological diagnoses, TNM classifications (8th edition of the AJCC staging system), and histological risk factors—lymphovascular invasion, spread through air spaces (STAS), and histological grade—were reviewed using hematoxylin and eosin (H&E)‐stained slides. Two pulmonary pathologists (Y.E.S. and M.K.) independently evaluated the cases, resolving any discrepancies through consensus. This study was approved by the Institutional Review Board of Seoul St. Mary's Hospital, Catholic University of Korea (KC22SISI0845).

### 2.2 Sample Preparation, Targeted Next‐Generation Sequencing, and Data Processing

DNA and RNA were extracted from formalin‐fixed, paraffin‐embedded (FFPE) tumor samples using the RecoverAll Total Nucleic Acid Isolation Kit for FFPE (Thermo Fisher Scientific, Waltham, MA, USA). Nucleic acid quality and quantity were assessed via spectrophotometry (NanoDrop, Thermo Fisher Scientific) and fluorometric quantification (Qubit, Thermo Fisher Scientific). Samples meeting quality thresholds underwent sequencing.

Targeted NGS was performed using the Oncomine Comprehensive Assay Plus DNA Panel (Thermo Fisher Scientific), which detects somatic mutations, copy number variations, and gene fusions across 501 cancer‐related genes, including key oncogenes and tumor suppressor genes such as EGFR, TP53, PIK3CA, AR, and MDM2. Libraries were prepared using Ion AmpliSeq technology, and sequencing was conducted on the Ion Torrent platform. Bioinformatic analysis utilized Ion Reporter software, with filtering based on allele frequency (≥ 2%), coverage (≥ 500×), and quality metrics. Variants were annotated using ClinVar, COSMIC, and dbSNP databases.

### 2.3 Immunohistochemistry

FFPE tissue sections were cut at a thickness of 4 μm and mounted on slides for immunohistochemistry (IHC) analysis. P53 IHC was performed using the DO‐7 monoclonal antibody (Roche Diagnostics, Basel, Switzerland), with a ready‐to‐use formulation, and detected using the UltraView Universal DAB Detection Kit (Ventana Medical Systems, Tucson, AZ, USA). EGFR IHC was conducted using the EGFR.113 mouse monoclonal antibody (Leica Biosystems, Wetzlar, Germany) at a 1:50 dilution, with the Bond Polymer Refine Detection Kit on a BOND‐III automated stainer (Leica Biosystems, Wetzlar, Germany).

P53 IHC results were categorized based on the percentage of positive cells, excluding null‐type cases (< 1% positive staining). EGFR IHC was scored using the histochemical scoring (*H*‐score) method, where staining intensity (*i*) was defined as 0 = no staining; 1 = weak; 2 = moderate; 3 = strong, and the percentage of stained cells at each intensity level (*Pi*) was recorded. The *H*‐score was calculated as Σ (*i* × *Pi*), resulting in scores from 0 to 300. For surgical specimens, IHC analysis was conducted on a single representative slide, which was also used for DNA extraction and NGS analysis. For biopsy specimens, IHC evaluation was performed across all available slides to account for tumor heterogeneity. Two pulmonary pathologists (Y.E.S. and M.K.) independently reviewed results, resolving discrepancies by consensus.

### 2.4 Statistical Analysis

Statistical analyses were conducted using R software (version 4.3.0; R Foundation, Vienna, Austria). Survival analyses, including Kaplan–Meier (KM) curves and Cox proportional hazards models, were used to assess OS and disease‐free survival (DFS). The log‐rank test evaluated survival differences, and hazard ratios (HRs) with 95% confidence intervals (CIs) were calculated.

Associations between genetic alterations (e.g., TP53 mutations, EGFR amplification) and clinicopathological factors (e.g., T stage, lymphovascular invasion, histological grade) were analyzed using chi‐square or Fisher's exact tests.

For IHC analysis, receiver operating characteristic (ROC) curve analysis determined the predictive power of p53 and EGFR IHC for genetic alterations. Optimal cutoff values were identified using Youden's index, and the area under the curve (AUC) quantified predictive accuracy. To assess statistical reliability, 95% CIs were calculated for all estimates. Statistical significance was set at *p* < 0.05.

To ensure the adequacy of our sample size, we performed a post hoc power calculation using the Schoenfeld formula for survival analysis. Assuming a HR of 2.0, an alpha of 0.05, and a power of 80%, we estimated that a minimum of 16 recurrence or death events would be required to achieve sufficient statistical power. In this study, we observed 108 recurrence or death events, indicating that our sample size was sufficient for robust statistical analysis.

## 3 Results

### 3.1 Clinicopathological Characteristics of Patients

A total of 424 patients with EGFR‐mutated NSCLC were included (Table 1). The cohort comprised 277 females (65.3%) and 147 males (34.7%), with a median age of 66 years (IQR: 59–72 years). Most patients were never smokers (71.5%), and adenocarcinoma was the predominant histological type (97.9%). Surgical specimens were analyzed in 329 cases (77.6%), with the remaining 95 cases (22.4%) being biopsy samples. Stage distribution included 61.8% of patients in Stage 1, 4.5% in Stage 2, 9.7% in Stage 3, and 24.1% in Stage 4. The most common EGFR mutation types were L858R (46.5%) and E19del (40.8%), with other mutations accounting for the rest.

**TABLE 1:** Clinicopathological characteristics of the enrolled patients with EGFR mutations (*n* = 424).

Characteristic	No. of patients (%)
Sex	
Female	277 (65.3%)
Male	147 (34.7%)
Age	
Smoking	
Never smoker	303 (71.5%)
Smoker	121 (28.5%)
Ex‐smoker	
Current smoker	
Diagnosis	
Adenocarcinoma	415 (97.9%)
Adenosquamous carcinoma	3 (0.7%)
Combined small cell carcinoma and adenocarcinoma	3 (0.7%)
Mixed invasive mucinous and nonmucinous adenocarcinoma	2 (0.5%)
Pleomorphic carcinoma	1 (0.2%)
Specimen type	
Biopsy	95 (22.4%)
Surgery	329 (77.6%)
Stage	
1	262 (61.8%)
2	19 (4.5%)
3	41 (9.7%)
4	102 (24.1%)
EGFR mutation type	
L858R	197 (46.5%)
E19del	173 (40.8%)
E20ins	15 (3.5%)
G719	15 (3.5%)
L861Q	13 (3.1%)
L747P	4 (0.9%)
G719A, L861Q	2 (0.5%)
S768I, L861Q	1 (0.2%)
S768I	1 (0.2%)
Other	3 (0.7%)
Surgical specimen	
T stage	
1	239 (73.3%)
2	64 (19.6%)
3	13 (4%)
4	10 (3.1%)
N stage	
0	266 (82.4%)
1	15 (4.6%)
2	37 (11.5%)
3	5 (1.5%)
Lymphovascular invasion	
Negative	191 (58.1%)
Present	138 (41.9%)
Spread through air space	
Negative	189 (79.7%)
Present	48 (20.3%)
Pleural invasion	
PL0	262 (79.9%)
PL1	33 (10.1%)
PL2	27 (8.2%)
PL3	6 (1.8%)
Histological grade	
G1	103 (31.7%)
G2	158 (48.6%)
G3	64 (19.7%)

### 3.2 Distribution of Concurrent Gene Alterations

**FIGURE 1:**
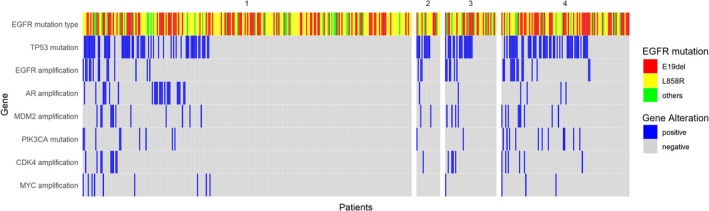
Concurrent genetic alterations, including TP53 mutations, EGFR amplification, AR amplification, and PIK3CA mutations, across different stages in patients with EGFR mutations. Each bar represents a patient, with colors—red, yellow, and green—indicating specific EGFR mutation types.

Concurrent genetic alterations, including TP53 mutations, EGFR amplification, AR amplification, MDM2 amplification, and PIK3CA mutations, exhibited stage‐specific variability (Figure 1). TP53 mutations increased significantly from 21.37% in Stage 1 to 43.14% in Stage 4 (*p* < 0.001). EGFR amplification was more frequent in Stages 2–4 compared to Stage 1 (*p* = 0.005), while PIK3CA mutations were notably higher in Stage 4 (*p* = 0.015). AR, MDM2, CDK4, and MYC amplifications showed no stage‐specific differences (*p* > 0.05) (Table 2). The frequency of concurrent alterations did not significantly differ between EGFR mutation subtypes (L858R, E19del, and others; all *p* > 0.05) (Table S1). In addition, TP53 mutations were associated with a higher tumor mutation burden (TMB), with a trend toward increased mean TMB in TP53‐mutant cases compared to TP53 wild‐type cases (4.93 ± 2.32 vs. 4.28 ± 2.10, *p* = 0.066) (Figure S1).

**TABLE 2:** Proportion of gene alterations by stage.

Stage	TP53 mutation	EGFR amplification	AR amplification	MDM2 amplification	PIK3CA mutation	CDK4 amplification	MYC amplification
1	56/262 (21.37%)	14/262 (5.34%)	23/262 (8.78%)	12/262 (4.58%)	9/262 (3.44%)	9/262 (3.44%)	9/262 (3.44%)
2	9/19 (47.37%)	3/19 (15.79%)	1/19 (5.26%)	2/19 (10.53%)	1/19 (5.26%)	1/19 (5.26%)	0/19 (0%)
3	17/41 (41.46%)	6/41 (14.63%)	1/41 (2.44%)	4/41 (9.76%)	1/41 (2.44%)	4/41 (9.76%)	1/41 (2.44%)
4	44/102 (43.14%)	14/102 (13.73%)	4/102 (3.92%)	9/102 (8.82%)	11/102 (10.78%)	6/102 (5.88%)	3/102 (2.94%)
Total	126/424 (29.72%)	37/424 (8.73%)	29/424 (6.84%)	27/424 (6.37%)	22/424 (5.19%)	20/424 (4.72%)	13/424 (3.07%)
*p*	**< 0.001**	**0.005**	0.064	0.093	**0.015**	0.173	0.748

*Note:* Bold values indicate statistically significant differences (*p* < 0.05).

### 3.3 Prognostic Implications of Concurrent Alterations

**FIGURE 2:**
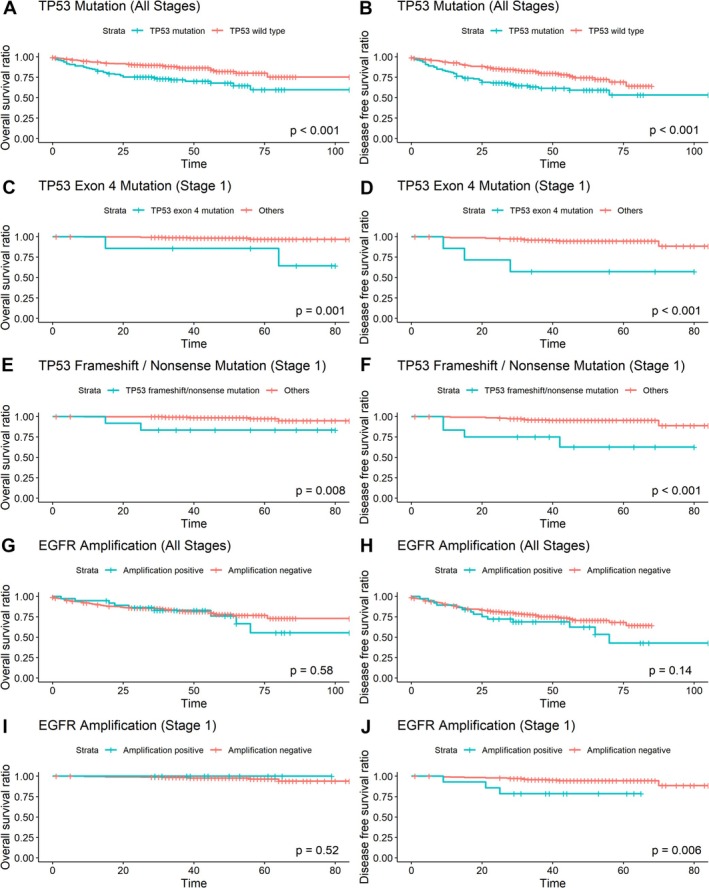
Kaplan–Meier (KM) survival curves for EGFR mutation‐positive lung cancer. (A, B) Overall survival (OS) and disease‐free survival (DFS) in all stages with concurrent TP53 mutations. (C, D) OS and DFS in Stage 1 with TP53 exon 4 mutations. (E, F) OS and DFS in Stage 1 with TP53 frameshift/nonsense mutations. (G, H) OS and DFS in all stages with EGFR amplification. (I, J) OS and DFS in Stage 1 with EGFR amplification.

Cox proportional hazards analysis revealed that TP53 mutations were associated with worse OS (HR: 2.33, *p* < 0.001) and DFS (HR: 2.09, *p* < 0.001) across all stages (Table S2). However, stage‐specific analyses showed no significant prognostic impact of TP53 mutations. EGFR amplification did not correlate with OS in the overall cohort but was significantly associated with worse DFS in Stage 1 (HR: 4.94, *p* = 0.013). Kaplan–Meier curves (Figure 2) highlighted significant survival differences in Stage 1, where TP53 exon 4 mutations were linked to worse OS (HR: 10.08, 95% CI: 1.85–54.97, *p* = 0.008) and DFS (HR: 9.68, 95% CI: 2.72–34.42, *p* < 0.001). Frameshift/nonsense TP53 mutations were also associated with worse OS (HR: 6.93, 95% CI: 1.32–36.44, *p* = 0.022) and DFS (HR: 7.92, 95% CI: 2.55–24.62, *p* < 0.001) in Stage 1.

### 3.4 Association of Genetic Alterations With Clinicopathological Factors

TP53 mutations correlated with advanced T stage (*p* = 0.012), N stage (*p* = 0.002), lymphovascular invasion (*p* < 0.001), STAS (*p* < 0.001), pleural invasion (*p* = 0.005), and high histological grade (*p* < 0.001). EGFR amplification was significantly associated with lymphovascular invasion (*p* < 0.001), STAS (*p* = 0.012), and high histological grade (*p* < 0.001). Smoking status was not associated with either TP53 mutations (*p* = 0.549) or EGFR amplification (*p* = 1.000) (Table 3).

**TABLE 3:** Association of TP53 mutation and EGFR amplification with clinicopathological risk factors.

	TP53 mutation		EGFR amplification	
	*N*	*p*	*N*	*p*
T stage				
T1	52/239 (21.8%)	**0.012**	13/239 (5.4%)	0.245
T2	21/64 (32.8%)		6/64 (9.4%)	
T3	5/13 (38.5%)		1/13 (7.7%)	
T4	6/10 (60.0%)		2/10 (20.0%)	
N stage				
> N1	25/57 (43.9%)	**0.002**	9/57 (15.8%)	**0.0142**
Lymphovascular invasion				
Present	54/138 (39.1%)	**< 0.001**	18/138 (13.0%)	**< 0.001**
Spread through airspace				
Present	22/48 (45.8%)	**< 0.001**	7/48 (14.6%)	**0.012**
Pleural ivnasion				
Present	26/66 (39.4%)	**0.005**	8/66 (12.1%)	0.091
Histological grade				
Grade 3	30/64 (46.9%)	**< 0.001**	12/64 (18.8%)	**< 0.001**
Smoking				
Present	39/121 (32.2%)	0.549	11/121 (9.1%)	1.000

*Note:* Bold values indicate statistically significant differences (*p* < 0.05).

### 3.5 Correlation of Genetic Alterations With Immunohistochemistry

**FIGURE 3:**
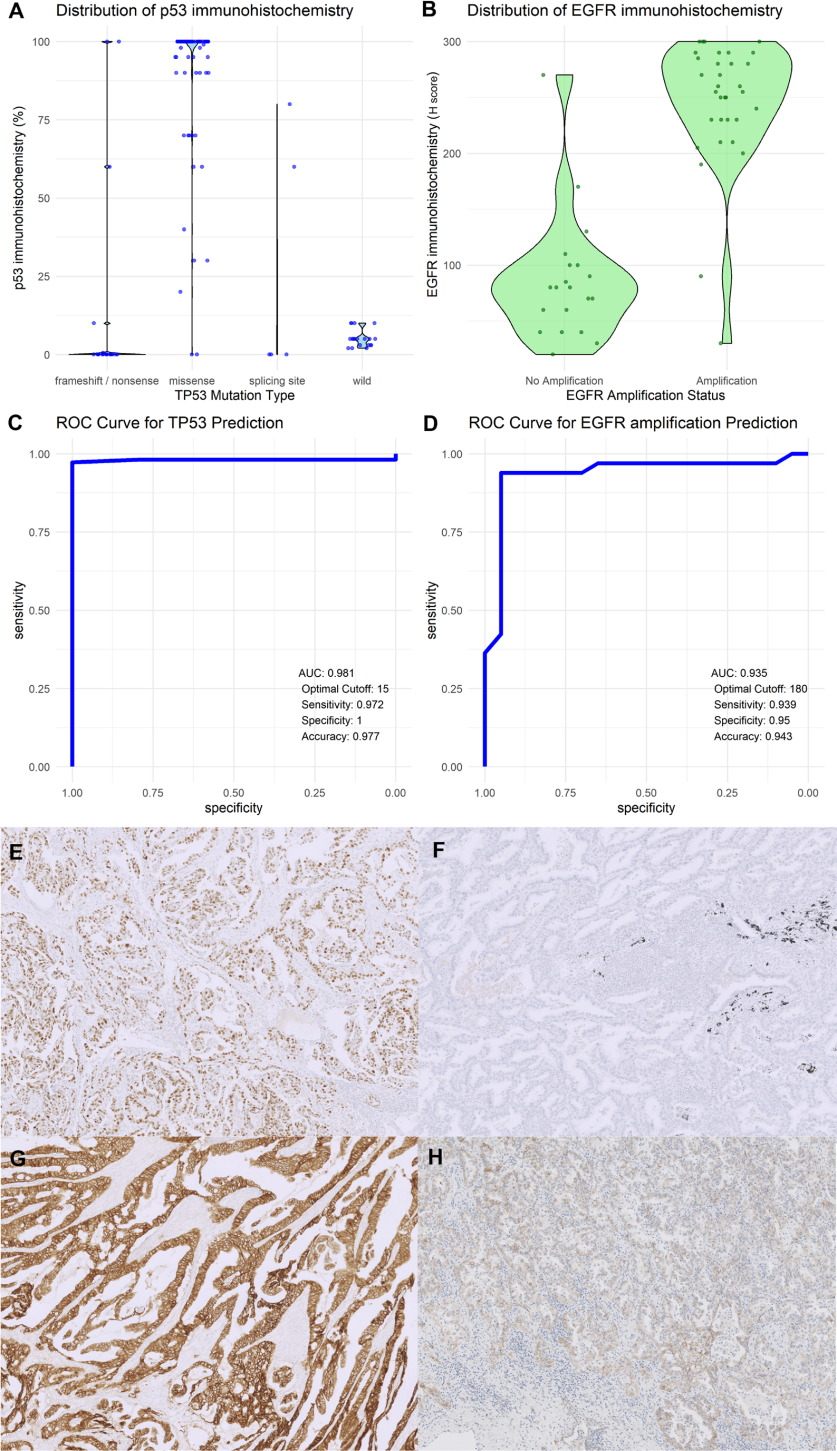
Immunohistochemistry (IHC) staining patterns of p53 and EGFR, and their ability to predict gene alterations. (A) Violin plot of p53 IHC staining proportion across mutation types. (B) Violin plot of EGFR IHC *H*‐scores across amplification status. (C) Receiver operating characteristic (ROC) curve of p53 IHC for TP53 mutation prediction. (D) ROC curve of EGFR IHC for EGFR amplification prediction. (E–H) Representative images showing p53 IHC overexpression (E) and null type (F), as well as EGFR IHC with *H*‐score 300 (G) and *H*‐score 100 (H).

IHC patterns varied according to TP53 mutation type (Figure 3). Null staining (0%) was predominant in frameshift/nonsense mutations, while missense mutations predominantly exhibited overexpression (90%–100%), with intermediate patterns (15%–90%) also observed. Wild‐type TP53 typically showed staining intensities between 1% and 10%.

EGFR amplification correlated with *H*‐scores. Cases without amplification generally scored 0–100, while amplified cases scored 200–300. ROC curve analysis identified cutoffs of 15% for TP53 IHC (AUC = 0.981) and *H*‐score ≥ 180 for EGFR IHC (AUC = 0.935) as highly predictive of TP53 mutations and EGFR amplification, respectively. Representative images illustrate p53‐positive/null cases and EGFR‐amplified/nonamplified cases (Figure 3).

## 4 Discussion

Extensive research has highlighted the role of co‐occurring genetic alterations in EGFR‐mutated NSCLC. Among these, TP53 mutations are the most common, followed by EGFR amplification, RB1, PIK3CA, and MYC [13]. In this study, targeted sequencing identified TP53 mutations as the most frequent alteration, with EGFR amplification as the second most common, consistent with prior findings. Numerous studies have underscored the association of TP53 mutations with poor prognosis and reduced responsiveness to TKIs in advanced NSCLC [14]. However, the complexity of TP53 mutation classifications has led to inconsistent conclusions regarding their prognostic implications [14].

Our findings showed that TP53 mutations were more frequent in advanced stages and were associated with worse OS and DFS across all stages. Interestingly, stage‐specific analyses revealed no significant prognostic impact of TP53 mutations, suggesting that their association with poor outcomes may be mediated through advanced disease. Previous studies have demonstrated a correlation between TP53 mutations and increased TMB [15]. In this study, TP53‐mutant tumors exhibited a trend toward higher TMB compared to TP53 wild‐type tumors, although the difference did not reach statistical significance (*p* = 0.066). While not a primary focus of this study, this finding warrants further investigation in larger cohorts to elucidate the potential interaction between TP53 mutations and genomic instability in NSCLC.

Several studies have emphasized the importance of categorizing TP53 mutations, such as by exon or mutation type, to better understand their prognostic impact. Without such detailed classification, the overall prognostic significance of TP53 mutations in NSCLC may seem inconsistent [16, 17]. TP53 mutations are frequently observed in exons 4–8, with these regions being the most affected [18]. One study identified mutations in exons 4–7 as independent prognostic factors for worse PFS and OS in advanced NSCLC patients treated with EGFR‐TKIs [19]. In contrast, another study found that exon 7 mutations were specifically associated with significantly worse outcomes compared to wild‐type TP53 [20]. Furthermore, a different study reported that mutations in exons 4, 6, and unknown/multiple regions were linked to worse outcomes than those in exons 5, 7, 8, and 9 [18].

In our study, across all stages, mutations in exons 4, 5, and 6 were associated with worse OS, while mutations in exons 4 and 6 were linked to worse DFS (Table S3). Although TP53 mutations did not show overall prognostic significance in Stage 1 patients, further analysis revealed that exon 4 mutations, as well as frameshift and nonsense mutations, were significantly associated with poorer outcomes. This highlights the necessity of considering both the specific exon location and mutation type to fully understand the prognostic significance of TP53 mutations.

EGFR amplification has been linked to poor prognosis in advanced NSCLC, particularly in metastatic settings. For instance, EGFR amplification was associated with poorer OS in patients with leptomeningeal metastases after progression on first‐generation and third‐generation TKIs [21]. Additionally, EGFR amplification was identified as a frequent coalteration, occurring in up to 40% of cases at the time of leptomeningeal progression, with significantly higher rates compared to initial diagnoses (*p* < 0.01). Multivariate analyses have further suggested its role as an independent prognostic factor for shorter OS in these patients [22]. However, some reports, such as a 2022 prospective study [23], showed no significant prognostic impact in multivariate analysis for first‐line TKI‐treated advanced NSCLC (*p* = 0.321), though EGFR amplification was linked to a higher risk of brain metastasis (*p* = 0.047).

Despite these insights, most of the current literature focuses on advanced or metastatic stages, leaving a noticeable gap in the understanding of EGFR amplification's role in early‐stage NSCLC. In our study, EGFR amplification was associated with shorter OS and DFS in Stage 4 and worse DFS in Stage 1. This suggests that its prognostic significance may extend to earlier stages, warranting further investigation. These results also highlight the variability in EGFR amplification's impact across different stages of NSCLC.

The observed associations between TP53 mutations and EGFR amplifications with key clinicopathological risk factors (Table 3) provide insight into their potential prognostic impact. Both genetic alterations were significantly linked to features indicative of tumor aggressiveness, such as advanced T stage, lymphovascular invasion, spread through airspace (STAS), and high histological grade. These associations underline their role in driving aggressive tumor biology and poorer clinical outcomes.

These results emphasize the importance of considering the combined effects of genetic alterations and clinicopathological features in understanding disease prognosis and tailoring personalized treatment strategies. The strong association of TP53 mutations and EGFR amplifications with high‐risk pathological factors supports their value as biomarkers for identifying patients with more aggressive disease who may benefit from intensified therapeutic approaches.

Previous studies, such as the one by Sung et al. have demonstrated that p53 IHC can correlate well with mutation patterns in cancers like breast, ovarian, and colorectal cancers when classified into overexpression, null type, and usual patterns [24]. However, similar studies in lung cancer are relatively limited. In our study, we observed that frameshift/nonsense mutations predominantly displayed a null type staining pattern, while missense mutations were mostly associated with overexpression patterns. Interestingly, expression levels ranging from 20% to 90% were also occasionally observed, and wild‐type p53 was almost exclusively stained between 1% and 10%.

Using ROC curve analysis, we identified a 15% cutoff that effectively predicted mutation status across all types of p53 mutations. Notably, null‐type staining was strongly predictive of frameshift/nonsense mutations. For EGFR amplification, a correlation was observed between EGFR IHC *H*‐scores and amplification status. With a cutoff *H*‐score of 180, amplification status could be accurately predicted, as shown by an AUC of 0.936. These findings suggest that IHC can serve as a practical surrogate for genetic testing, particularly in early‐stage NSCLC where molecular testing may not always be feasible. This approach could optimize diagnostic workflows and inform treatment decisions in resource‐limited settings.

This study's strengths include its large cohort size and comprehensive stage‐specific analyses of TP53 mutations and EGFR amplification. Notably, it addresses the underexplored prognostic implications of these alterations in early‐stage NSCLC. However, several limitations should be acknowledged. First, this study is a single‐institution, retrospective analysis, which may introduce selection bias and limit the generalizability of our findings. Given that our institution is a high‐volume center for early‐stage lung cancer treatment, there is a predominance of Stage 1 patients in our cohort, which may influence survival outcomes. However, we performed stage‐specific survival analyses and adjusted for stage in multivariate Cox models, confirming that TP53 mutations and EGFR amplification were independent prognostic factors. Second, the lack of an external validation cohort restricts the reproducibility of our results, particularly in the establishment of IHC cutoff values for TP53 and EGFR amplification. Third, potential interobserver variability in IHC interpretation could affect consistency in biomarker evaluation. While internal validation was performed using ROC analysis to define optimal cutoff values, additional external validation is warranted to confirm their clinical applicability. Furthermore, the relatively low number of recurrence and death events in early‐stage cases may have impacted the survival analysis, though a post hoc power calculation confirmed the adequacy of our sample size. Future multicenter prospective studies with external validation are necessary to address these limitations and further establish the prognostic utility of TP53 mutations and EGFR amplification in early‐stage NSCLC.

## 5 Conclusion

In early‐stage lung cancer, where surgical resection is typically considered curative, identifying patients at higher risk of recurrence is critical. Our study suggests that TP53 mutations, particularly frameshift, nonsense, or exon 4 mutations, as well as EGFR amplification, may be associated with an increased risk of recurrence. For EGFR‐positive patients, selectively offering adjuvant therapies to those at higher risk could provide better treatment options and improve outcomes. Further research is essential to validate these findings and explore their broader clinical implications.

## Author Contributions

All authors had full access to the data in the study and take responsibility for the integrity of the data and the accuracy of the data analysis. Conceptualization: G.S.P., K.Y.L., and Y.E.S.; methodology: G.S.P. and K.Y.L.; data curation: M.K. and S.W.M.; formal analysis: M.K.; writing – original draft preparation: M.K.; writing – review and editing: Y.E.S. and K.Y.L.; visualization: Y.E.S. and M.K.; supervision: Y.E.S.

## Conflicts of Interest

The authors declare no conflicts of interest.

## Supporting information


**Figure S1.** Comparison of Tumor Mutation Burden (TMB) by TP53 Mutation Status.

Data S1.

## Data Availability

The data that support the findings of this study are available from the corresponding author upon reasonable request.
