# Supplementary figures and images for: Role of TP53 Mutations and EGFR Amplification in Risk Stratification of Early‐Stage EGFR‐Mutated Non‐Small Cell Lung Cancer With Immunohistochemistry as a Surrogate Marker
Source: Thorac Cancer. 2025 Apr 1;16(7):e70058. doi: 10.1111/1759-7714.70058 (PMC11959145; doi:10.1111/1759-7714.70058)

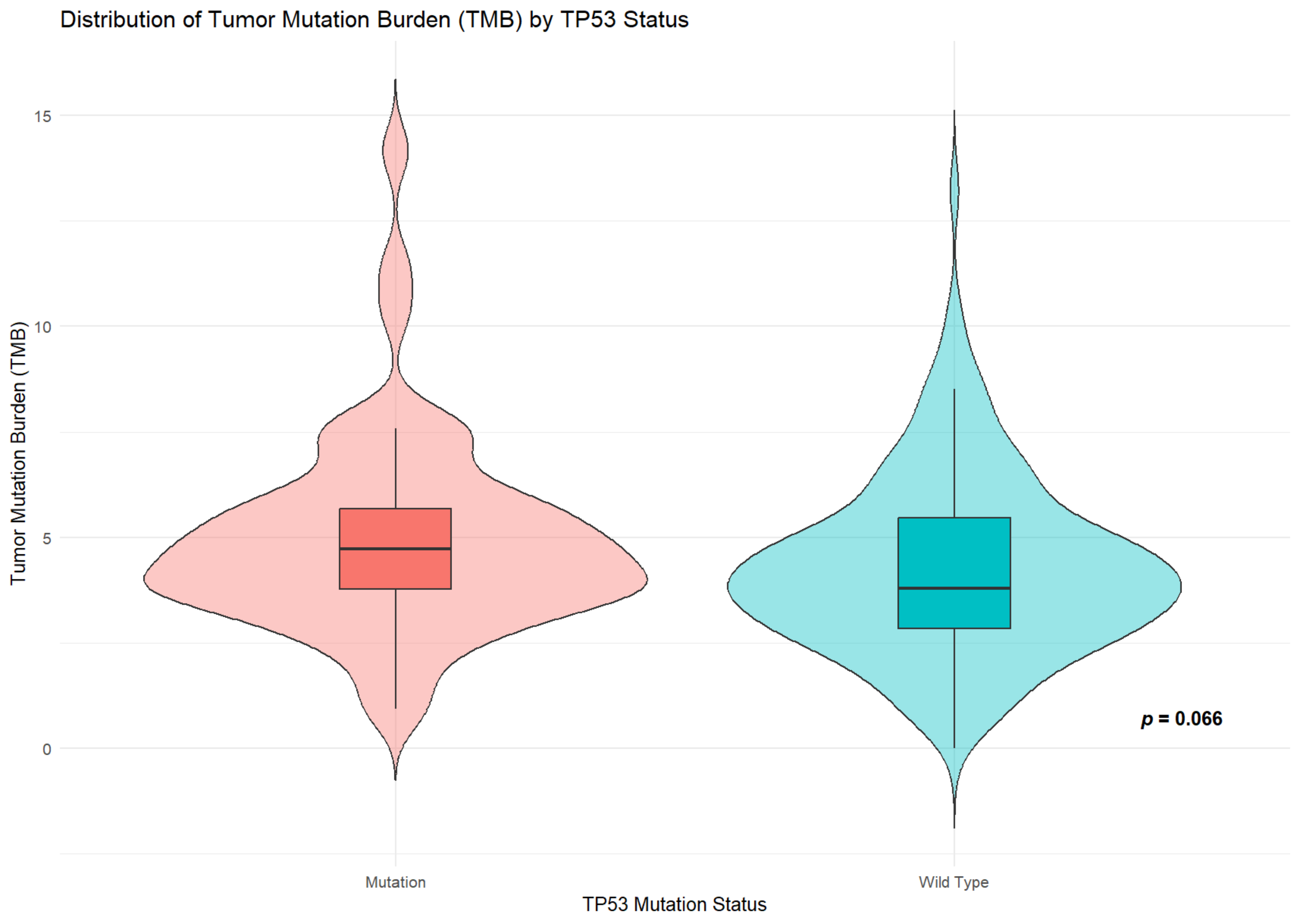

Supplement: Supplementary file 1 — Figure S1. Comparison of Tumor Mutation Burden (TMB) by TP53 Mutation Status. [file TCA-16-e70058-s002.tiff]
