# Supplementary material for: Role of TP53 Mutations and EGFR Amplification in Risk Stratification of Early‐Stage EGFR‐Mutated Non‐Small Cell Lung Cancer With Immunohistochemistry as a Surrogate Marker
Source: Thorac Cancer. 2025 Apr 1;16(7):e70058. doi: 10.1111/1759-7714.70058 (PMC11959145; doi:10.1111/1759-7714.70058)
Supplement: Supplementary file 2 — Data S1. [file TCA-16-e70058-s001.docx]

**Supplementary Table 1**. Frequency of Concurrent Gene Alteration by EGFR Mutation Type

| **EGFR mutation type** | **TP53**  **mutation** | **EGFR amplification** | **AR**  **amplification** | **MDM2 amplification** | **PIK3CA**  **mutation** | **CDK4 amplification** | **MYC amplification** |
| --- | --- | --- | --- | --- | --- | --- | --- |
| **E19del** | 48 / 173 (27.75%) | 17 / 173 (9.83%) | 12 / 173 (6.94%) | 11 / 173 (6.36%) | 12 / 173 (6.94%) | 8 / 173 (4.62%) | 7 / 173 (4.05%) |
| **L858R** | 58 / 197 (29.44%) | 15 / 197 (7.61%) | 16 / 197 (8.12%) | 13 / 197 (6.6%) | 6 / 197 (3.05%) | 10 / 197 (5.08%) | 4 / 197 (2.03%) |
| **Other mutation** | 20 / 54 (37.04%) | 5 / 54 (9.26%) | 1 / 54 (1.85%) | 3 / 54 (5.56%) | 4 / 54 (7.41%) | 2 / 54 (3.7%) | 2 / 54 (3.7%) |
| Total | 126/424 (29.72%) | 37/424 (8.73%) | 29/424 (6.84%) | 27/424 (6.37%) | 22/424 (5.19%) | 20/424 (4.72%) | 13/424 (3.07%) |
| p-value | 0.4243 | 0.7452 | 0.2699 | 0.962 | 0.1779 | 0.9125 | 0.5107 |

**Supplementary Table 2**. Hazard Ratios for Concurrent Genetic Alterations Across Stages in EGFR-Positive Lung Cancer: Cox Proportional Hazards Model Analysis

1. Overall survival

| **Stage** | **TP53**  **mutation** | **EGFR**  **amplification** | **AR**  **amplification** | **MDM2**  **amplification** | **PIK3CA**  **Mutation** | **CDK4**  **amplification** | **MYC**  **amplification** |
| --- | --- | --- | --- | --- | --- | --- | --- |
| 1 | 2.748  (0.614 - 12.300)  (p=0.186) | 0 (0 - inf) (p=0.998) | 0 (0 - inf) (p=0.999) | 4.604  (0.544 - 38.998) (p=0.161) | 4.271  (0.512 - 35.596) (p=0.18) | 0 (0 - inf) (p=0.999) | 0 (0 - inf) (p=0.999) |
| 2 | 2.385  (0.215 - 26.44)  (p=0.479) | 0 (0 - inf) (p=0.998) | 0 (0 - inf) (p=0.999) | NA | 0 (0 - inf) (p=0.999) | 0 (0 - inf) (p=0.999) | NA |
| 3 | 1.504  (0.376 - 6.017) (p=0.564) | 4.244  (1.009 - 17.86)  **(p=0.049)** | 0 (0 - inf) (p=0.999) | 3.17  (0.634 - 15.85)  (p=0.16) | 0 (0 - inf) (p=0.999) | 2.83  (0.57 - 14.044)  (p=0.203) | 14.707  (1.509 - 143.376) (p=0.021) |
| 4 | 1.303  (0.790 - 2.148) (p=0.3) | 0.427  (0.183 - 0.998)  **(p=0.049)** | 0.583  (0.142 - 2.395)  (p=0.454) | 0.829  (0.299 - 2.299) (p=0.719) | 0.672  (0.288 - 1.569) (p=0.358) | 0.576  (0.14 - 2.368)  (p=0.445) | 1.052  (0.256 - 4.327)  (p=0.944) |
| Total | 2.33  (1.502 - 3.615) **(p < 0.001**) | 1.217  (0.608 - 2.437) p=0.579 | 0.3455  (0.085 - 1.408) p=0.138 | 1.719  (0.7883 - 3.75) p=0.173 | 1.670  (0.7675 - 3.635) p=0.196 | 1.114  (0.407 - 3.046) p=0.834 | 1.245  (0.393 - 3.947) p=0.710 |

1. Disease free survival

| **Stage** | **TP53**  **mutation** | **EGFR**  **amplification** | **AR**  **amplification** | **MDM2**  **amplification** | **PIK3CA**  **Mutation** | **CDK4**  **amplification** | **MYC**  **amplification** |
| --- | --- | --- | --- | --- | --- | --- | --- |
| 1 | 2.469  (0.894 - 6.814)  (p=0.081) | 4.945  (1.394 - 17.538)  **(p=0.013)** | 0 (0 - inf) (p=0.998) | 3.51  (0.790 - 15.596)  (p=0.099) | 1.863  (0.245 - 14.149)  (p=0.547) | 1.812  (0.239 - 13.758)  (p=0.565) | 0 (0 - inf) (p=0.998) |
| 2 | 1.816  (0.481 - 6.862)  (p=0.379) | 2.955  (0.557 - 15.673)  (p=0.203) | 0 (0 - inf) (p=0.998) | 1.817  (0.210 - 15.706)  (p=0.587) | 2.385  (0.286 - 19.904)  (p=0.422) | 0 (0 - inf) (p=0.998) | NA |
| 3 | 0.738  (0.297 - 1.831)  (p=0.512) | 1.094  (0.321 - 3.731)  (p=0.886) | 4.398  (0.550 - 35.192)  (p=0.163) | 1.539  (0.452 - 5.238)  (p=0.491) | 1.433  (0.190 - 10.804)  (p=0.727) | 1.504  (0.442 - 5.117)  (p=0.514) | 3.829  (0.485 - 30.251)  (p=0.203) |
| 4 | 1.303  (0.790 - 2.148)  (p=0.300) | 0.427  (0.183 - 0.998)  **(p=0.049)** | 0.583  (0.142 - 2.395)  (p=0.454) | 0.829  (0.299 - 2.299)  (p=0.719) | 0.672  (0.288 - 1.569)  (p=0.358) | 0.576  (0.140 - 2.368)  (p=0.445) | 1.052  (0.256 - 4.327)  (p=0.944) |
| Total | 2.094  (1.430 - 3.066)  **(p < 0.001)** | 1.526  (0.87 - 2.679)  (p=0.141) | 0.363  (0.115 - 1.144)  (p=0.084) | 1.856  (0.964 - 3.575)  (p=0.064) | 1.615  (0.815 - 3.199)  (p=0.169) | 1.248  (0.547 - 2.845) (p=0.599) | 0.878  (0.279 - 2.769) (p=0.825) |

**Supplementary Table 3.** Hazard Ratios for TP53 Mutations by Exon Across Stages in EGFR-Positive Lung Cancer

1. Overall survival

| **Stage** | **TP53 exon 4** | **TP53 exon 5** | **TP53 exon 6** | **TP53 exon 7** | **TP53 exon 8** | **TP53 other** | **TP53 total** |
| --- | --- | --- | --- | --- | --- | --- | --- |
| 1 | 10.082 (1.849 - 54.969) **(p=0.008)** | 0 (0 - inf) (p=0.998) | 0 (0 - inf) (p=0.998) | 0 (0 - inf) (p=0.998) | 3.391 (0.404 - 28.44)  (p=0.26) | 0 (0 - inf) (p=0.998) | 2.748 (0.614 - 12.300)  (p=0.186) |
| 2 | NA | 5.622 (0.508 - 62.192)  (p=0.159) | 0 (0 - inf) (p=0.999) | 0 (0 - inf) (p=0.999) | 3.562 (0.301 - 42.216)  (p=0.314) | NA | 2.385 (0.215 - 26.44)  (p=0.479) |
| 3 | NA | 0 (0 - inf) (p=0.999) | 3.088 (0.37 - 25.743)  (p=0.297) | 5.133 (1.218 - 21.625)  **(p=0.026)** | 0 (0 - inf) (p=0.998) | 0 (0 - inf) (p=0.999) | 1.504 (0.376 - 6.017) (p=0.564) |
| 4 | 2.146 (0.667 - 6.912)  (p=0.201) | 2.251 (0.962 - 5.265)  (p=0.061) | 0.896 (0.467 - 1.72)  (p=0.742) | 0.636 (0.198 - 2.04)  (p=0.446) | 1.458 (0.525 - 4.051)  (p=0.469) | 1.574 (0.383 - 6.47)  (p=0.529) | 1.303 (0.790 - 2.148) (p=0.3) |
| Total | 2.557 (1.03 - 6.348)  **(p=0.043)** | 2.383 (1.096-5.179)  **(p=0.028)** | 2.639 (1.427 - 4.881)  **(p=0.002)** | 1.245 (0.542 - 2.863)  (p=0.605) | 1.191 (0.518 - 2.739)  (p=0.68) | 0.887 (0.218 - 3.611)  (p=0.867) | 2.33 (1.502 - 3.615) **(p < 0.001**) |

1. Disease Free Survival

| **Stage** | **TP53 exon 4** | **TP53 exon 5** | **TP53 exon 6** | **TP53 exon 7** | **TP53 exon 8** | **TP53 other** | **TP53 total** |
| --- | --- | --- | --- | --- | --- | --- | --- |
| 1 | 9.68 (2.722 - 34.416)  **(p < 0.001)** | 0 (0 - inf) (p=0.998) | 2.657 (0.349 - 20.242)  (p=0.346) | 0 (0 - inf) (p=0.998) | 3.22 (0.726 - 14.275)  (p=0.124) | 0 (0 - inf) (p=0.997) | 2.469 (0.894 - 6.814)  (p=0.081) |
| 2 | NA | 0.791 (0.099 - 6.354)  (p=0.826) | 2.501 (0.482 - 12.968)  (p=0.275) | 8.487 (0.769 - 93.653)  (p=0.081) | 0.717 (0.089 - 5.749)  (p=0.754) | NA | 1.816 (0.481 - 6.862)  (p=0.379) |
| 3 | NA | 0 (0 - inf) (p=0.997) | 0.907 (0.12 - 6.852)  (p=0.924) | 4.054 (1.454 - 11.305)  **(p=0.007)** | 0 (0 - inf) (p=0.998) | 0.538 (0.072 - 4.015)  (p=0.546) | 0.738 (0.297 - 1.831)  (p=0.512) |
| 4 | 2.146 (0.667 - 6.912)  (p=0.201) | 2.251 (0.962 - 5.265)  (p=0.061) | 0.896 (0.467 - 1.72)  (p=0.742) | 0.636 (0.198 - 2.04)  (p=0.446) | 1.458 (0.525 - 4.051)  (p=0.469) | 1.574 (0.383 - 6.47)  (p=0.529) | 1.303 (0.790 - 2.148)  (p=0.300) |
| Total | 2.763 (1.211 - 6.303)  **(p=0.016)** | 1.648 (0.766 - 3.547)  (p=0.201) | 2.544 (1.472 - 4.396)  **(p=0.001)** | 1.515 (0.765 - 3.000)  (p=0.233) | 1.043 (0.484 - 2.245)  (p=0.914) | 0.942 (0.299 - 2.968)  (p=0.918) | 2.094 (1.430 - 3.066)  **(p < 0.001)** |

**Supplementary Table 4.** Hazard Ratios for TP53 Mutation Types Across Stages in EGFR-Positive Lung Cancer

1. Overall survival

| **Stage** | **TP53 frameshift & nonsense mutation** | **TP53 missense mutation** | **TP53 splice site mutation** | **TP53 total** |
| --- | --- | --- | --- | --- |
| 1 | 6.925 (1.316 - 36.437)  **(p=0.022)** | 0.956 (0.115 - 7.947)  (p=0.967) | 0 (0 - inf) (p=0.999) | 2.748 (0.614 - 12.300)  (p=0.186) |
| 2 | 0 (0 - inf) (p=0.999) | 3.358 (0.302 - 37.313)  (p=0.324) | NA | 2.385 (0.215 - 26.44)  (p=0.479) |
| 3 | 0 (0 - inf) (p=0.999) | 2.141 (0.535 - 8.564)  (p=0.282) | 0 (0 - inf) (p=0.999) | 1.504 (0.376 - 6.017) (p=0.564) |
| 4 | 1.283 (0.549 - 3.000)  (p=0.566) | 1.144 (0.680 - 1.926)  (p=0.612) | 19.304(2.298-162.149) (p=0.006) | 1.303 (0.790 - 2.148) (p=0.3) |
| Total | 1.827 (0.878 - 3.800)  (p=0.107) | 2.118 (1.342 - 3.343)  **(p=0.001)** | 1.155(0.16-8.319) (p=0.886) | 2.33 (1.502 - 3.615) **(p < 0.001**) |

1. Disease free survival

| **Stage** | **TP53 frameshift & nonsense mutation** | **TP53 missense mutation** | **TP53 splice site mutation** | **TP53** |
| --- | --- | --- | --- | --- |
| 1 | 7.921 (2.548 - 24.623)  **(p < 0.001)** | 0.863 (0.195 - 3.810)  (p=0.846) | 0 (0 - inf) (p=0.998) | 2.469 (0.894 - 6.814)  (p=0.081) |
| 2 | 0 (0 - inf) (p=0.999) | 1.179 (0.312 - 4.449)  (p=0.808) | NA | 1.816 (0.481 - 6.862)  (p=0.379) |
| 3 | 0.817 (0.109 - 6.106)  (p=0.844) | 0.854 (0.331 - 2.206)  (p=0.745) | 0 (0 - inf) (p=0.998) | 0.738 (0.297 - 1.831)  (p=0.512) |
| 4 | 1.283 (0.549 - 3.000)  (p=0.566) | 1.144 (0.680 - 1.926)  (p=0.612) | 19.304 (2.298 - 162.149)  **(p=0.006)** | 1.303 (0.790 - 2.148)  (p=0.300) |
| Total | 2.424 (1.329 - 4.421)  **(p=0.004)** | 1.747 (1.164 - 2.624)  **(p=0.007)** | 0.773 (0.108 - 5.545)  (p=0.798) | 2.094 (1.430 - 3.066)  **(p < 0.001)** |
